# Chemical-Class Submixture Screening Reveals Drivers of Endocrine Disruption in Personalized Human Blood POP Mixtures

**DOI:** 10.1021/acs.est.5c13521

**Published:** 2026-02-03

**Authors:** Denise Strand, Paula Pierozan, Luã Reis, Bo Lundgren, Jonathan W. Martin, Oskar Karlsson

**Affiliations:** † Science for Life Laboratory, Department of Environmental Science, Stockholm University, Stockholm 114 18, Sweden; ‡ Science for Life Laboratory, Biochemical and Cellular Assay unit, Dept. of Biochemistry and Biophysics, 7675 Stockholm University, Stockholm 106 91, Sweden

**Keywords:** persistent organic pollutants, mixture toxicity, endocrine disruption, steroidogenesis, high-content analysis

## Abstract

Multiple studies demonstrate mixture effects arising from the interactive toxicity of environmental chemicals in human blood, but identifying the main toxic drivers remains challenging. In a recent proof-of-principle *in vitro* study, we showed that personalized mixtures (PMs), reconstructed from 24 persistent organic pollutant (POPs) concentrations measured in individual blood samples from Swedish adults, induced unique interindividual effects on H295R cell viability and steroidogenesis. Here, we followed up by testing submixtures of four PMs (PM#3, PM#4, PC1-OC-Mix, and PC2-PFAS-Mix), separated by the chemical classes perfluoroalkyl substances (PFASs), organochlorine pesticides (OCPs), polychlorinated biphenyls (PCBs), and polybrominated diphenyl ethers (PBDEs). Submixtures of PFAS and OCPs induced significant effects on testosterone synthesis at low (1×) and medium (10×) concentrations, consistent with effects observed in the corresponding whole PMs, and were therefore likely the primary drivers of the whole-mixture effects on testosterone. Notably, some submixtures altered estradiol and testosterone levels in ways not observed in full PMs, suggesting antagonistic interactions across chemical classes when combined. Potential antagonistic interaction in more complex mixtures, independent of concentration, was also observed within OCP submixtures, as only the less complex OCP mixtures lacking DDE or DDT induced testosterone synthesis. For additional mechanistic insight, we expanded the H295R assay to include oxidative stress analyses, which revealed no effects from the PMs. RT-qPCR analysis showed downregulation of *CYP11A1* after exposure to PM#3 and PM#4 at high concentrations (100×), suggesting a feedback mechanism contributing to suppressed testosterone synthesis.

## Graphical abstract

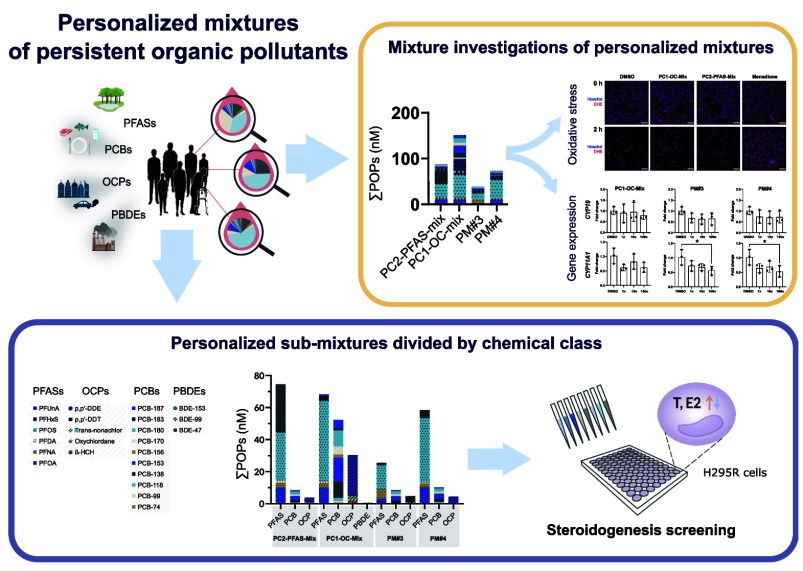

## Introduction

Endocrine disruption is a major mechanism by which environmental contaminants induce long-term adverse effects on human health. Persistent organic pollutants (POPs) are of particular concern due to their ubiquity, bioaccumulation, and persistence in the environment and long biological half-lives in human blood.^1^ Several POPs, including per- and polyfluoroalkyl substances (PFASs), polychlorinated biphenyls (PCBs), organochlorine pesticides (OCPs), and polybrominated diphenyl ethers (PBDEs), are suspected endocrine disruptors that can interfere with hormonal signaling, either via receptor binding or disruption of sex steroid hormone synthesis.^2,3^ Although many of these compounds have been restricted or banned,^1^ they can still leach from their original source materials and are frequently detected in drinking water, food, and human blood.^4,5^ Current chemical risk assessments most often rely on toxicological studies of individual compounds,^6^ but this approach overlooks the fact that humans are simultaneously and chronically exposed to multiple environmental contaminants from different chemical classes.^7^ The human chemical blood exposome varies widely between individuals due to geography, diet, personal care product use, and other lifestyle determinants.^8–10^

Whole-mixture toxicity studies using environmental extracts most accurately represent real-life exposure scenarios, but such approaches can be limited by unknown mixture compositions and low experimental sensitivity.^11^ Identifying which specific compounds or chemical classes that drive observed toxic effects remains a major challenge due to the complexity of environmental mixtures, and the presence of bioactive endogenous compounds in biological samples (e.g., blood) may interfere with interpretation.^12,13^ An alternative, more controlled approach involves reconstructing defined and realistic mixtures from pure compound stocks, enabling higher experimental sensitivity, the testing of wider concentrations ranges, and the ability to vary the mixture based on different time points, locations, or individuals.^10,14,15^

Given the central role of steroid hormone biosynthesis in endocrine regulation, *in vitro* models that quantify alterations in hormone production provide a mechanistically informative approach for assessing the endocrine-disruptive effects of complex chemical mixtures. The NCI-H295R steroidogenesis assay (OECD TG#456) is considered a sensitive and reproduceable method for assessing effects on estradiol and testosterone synthesis *in vitro*, with outcomes that are consistent with findings from *in vivo* studies.^58^ Several studies have reported steroidogenic effects of POP mixtures in H295R cells using extracts from matrices such as fish liver and defined mixtures.^16–19^ Recently, we conducted the first personalized mixture toxicology study using defined mixtures (PM) that matched real-world mixture compositions measured in individual people. These mixtures were rapidly and quantitatively reconstructed from small-volume stocks in multiwell plates using no-contact acoustic liquid handling.^10^ Significant effects on cell viability and on cellular estradiol and testosterone were observed in H295R cells exposed to these reconstructed PMs of POPs previously measured in the blood of individual Swedish adults.^3,10,20,21^ Importantly, the mixture effects were not always predictable from studies of individual POPs in the same system.^3^ To identify the toxicity drivers and underlying mechanisms in these real-world PMs, we hypothesized that no-contact liquid handling could again be used to reproducibly generate personalized submixtures of POPs, divided by chemical class, whose effects could be directly compared with those of the corresponding whole mixtures.^10^

The aim of the current study was therefore to investigate the drivers and mechanisms underlying the effects of personalized POP mixtures on the cell viability and sex hormone production in H295R cells. Four whole PMs (PM#3, PM#4, PC1-OC-Mix, and PC2-PFAS-Mix), examined in our recent study,^10^ were subdivided into chemical class-based submixtures and screened for their effects in H295R cells. High-content analysis (HCA) protocols were developed to expand the H295R steroidogenesis assay, enabling the mechanistic assessment of oxidative stress induction. This study provides further insight into the interactive endocrine-disruptive effects of real-world POP mixtures present in the blood of individual people.

## Methods

### Cell Culture

The human adrenocortical carcinoma cell line H295R was purchased from the American Type Culture Collection (ATCC, Manassas, VA, USA) and cultured according to OECD test guideline #456, with minor modifications as described in Strand et al. The H295R cell line has an endogenous production of steroid hormones. Briefly, cells were maintained in T75 flasks (Sarstedt, Nümbrecht, Germany) in a 1:1 Dulbecco’s modified Eagle medium and Nutrient Mixture F-12 (DMEM/F-12), supplemented with 1% ITS + premix containing insulin, human transferrin, and selenous acid (Corning Inc., Bedford, USA) and 2.5% NuSerum (Fisher Scientific, Waltham, MA, USA). Cultures were kept at 37 °C in a humidified atmosphere with 5% (v/v) CO_2_. Experiments were performed between passages 5 and 10 to ensure stable basal production of testosterone and estradiol.^3,22^

### Compounds and Mixture Generation Using No-Contact Liquid Handling

Twenty-four POPs belonging to classes PFAS, OCPs, PCBs, and PDBEs were selected based on their abundance in blood samples from a Swedish cohort enrolled in the Västerbotten intervention program (VIP).^20,21^ The effects of these compounds on cell viability and on testosterone and estradiol synthesis in H295R cells have previously been investigated both as individual compounds, tested in concentration–response (1 nM to 10 μM),^3^ and as personalized mixtures reconstructed based on blood levels from individuals.^10^ Here, selected personalized mixtures that induced endocrine-disrupting effects and decreased cell viability were further investigated as submixtures grouped by chemical class (Supplemental Table S1). All PFAS, PCBs, OCPs, and PBDEs were tested as submixtures to assess their contributions to overall bioactivity. This included two PMs from randomly selected individuals in the VIP cohort (PM#3 and PM#4), as well as two PMs strategically selected based on their scores in a principal component analysis (PCA), one with the POP-mixture variation predominantly driven by organochlorine (OC) compounds (PC1-OC-Mix) and the other primarily driven by PFAS (PC2-PFAS-Mix).^10^

Test compounds were obtained from Wellington Laboratories, Cambridge Isotope Laboratories, LGC standards, and Toronto Research Chemicals and are described in detail in Supplemental Table S2 of Strand et al 2024. Briefly, all compounds were dissolved in dimethyl sulfoxide (DMSO, CAS 67-68-5, Sigma-Aldrich, purity ≥99.9%) as previously described^3^ and transferred into an Echo-compatible 384-well source plate (Beckman Coulter Life Science, San José, USA). Treatment mixtures were generated in 96-well plates (Thermo Fisher Scientific Inc., Waltham, USA) from these compound stocks using the Echo 550 acoustic no-contact liquid handler (Beckman Coulter Life Sciences, San José, USA). Plates were sealed using a PlateLoc thermal microplate sealer (Agilent, Santa Clara, CA, USA) and stored at −20 °C unless used immediately.

### Effects of POP Mixtures on Testosterone and Estradiol Synthesis and Cell Viability in H295R Cells

To assess the impact of POP mixtures, grouped by chemical class, on testosterone and estradiol synthesis, an adapted version of the H295R steroidogenesis assay, based on the OECD test guideline (TG) #456, was used to allow for increased throughput, as previously described in detail.^3^ Briefly, 96-well tissue culture microplates (Sarstedt, Nümbrecht, Germany) were seeded overnight with 50,000 cells per well, excluding the outermost wells to minimize edge effects. Cells were then treated with POP mixtures, solvent control (DMSO 0.1%), or positive controls for steroidogenic suppression (prochloraz, 1 μM CAS 67747-09-5, 64947, LOT BCBT9975, purity 99.4%) or stimulation (forskolin, 10 μM, CAS 66575-29-9, F3917, LOT SLBZ0653, purity >98%), all diluted in cell culture medium. Each treatment condition was tested in six technical replicates. Cell viability was assessed after 48 h of exposure. Conditioned medium was collected and stored at −80 °C until quantification of testosterone and estradiol by ELISA.

### Analysis of Cell Viability by MTT

Following the end of treatment, exposed cells were incubated with 0.5 mg/mL of 3-(4,5-dimethylthiazol-2-yl)-2,5-diphenyltetrazolium bromide (MTT) solution, diluted in DMEM/F-12, at 37 °C for 1 h. Metabolically active cells reduce MTT to insoluble formazan crystals within the mitochondria. After removal of the excess MTT solution, the resulting formazan was dissolved in DMSO and shaken at 500 rpm for 10 min. Absorbance was then measured at 570 nm by using a SpectraMax i3x plate reader (Molecular Devices LLC, San José, USA). Cell viability was calculated as the percent of the solvent control after subtraction of the blank.

### Steroid Hormone Testosterone and Estradiol Quantification by ELISA

Quantification of testosterone and estradiol production by H295R cells was performed using ELISA kits (ADI-901-008, and ADI-901-065, ENZO Life Sciences Inc., Farmingdale, USA), according to the manufacturer’s instructions. From each well of the 96-well plate, 200 μL of conditioned medium was collected and analyzed for testosterone and estradiol content. Hormone concentrations (pg or ng/mL) were normalized to the solvent control to determine relative changes in steroid hormone synthesis in treated cells. In accordance with OECD guideline criteria, only experiments in which the positive controls, prochloraz and forskolin, produced the expected inhibitory or stimulatory effects on steroid hormone synthesis were included in the analysis. Specifically, prochloraz was required to reduce both hormone levels to ≤0.5-fold of the solvent control, while forskolin was required to induce a ≥1.5-fold increase in testosterone and a ≥7.5-fold increase in estradiol.

### Quantification of Gene Expression Using qRT-PCR

Cells were seeded at 750,000 cells per well and treated for 48 h in 6-well plates, with technical duplicates for each condition. After treatment, cells were rinsed with cold PBS, and total RNA was extracted using the RNeasy Mini kit (74104, Qiagen, Hilden, Germany) according to the manufacturer’s instructions. RNA concentration and purity were assessed using Nanodrop 1000 spectrophotometry (Thermo Scientific, Wilmington, DE, USA). Complementary DNA (cDNA) was synthesized from 1 μg of total RNA per sample using the maximum first-strand cDNA synthesis kit (K1641, Thermo Fisher, Vilnius, Lithuania). Quantitative real-time PCR (qRT-PCR) was performed using a CFX96 Real-Time PCR Detection System (Bio-Rad, Singapore) with 4 μL of cDNA and 6 μL of PCR mix containing Maxima SYBR Green PCR Master Mix (K022, Thermo Fisher, Vilnius, Lithuania). Primer sequences used were as follows: for *CYP11A1*, Fwr GGAGTCCTGTTGAAGAAGTCGG and Rev ACGAAGTCCCGAGACACTGC and for *CYP19A1* (*aromatase*), Fwr AAGACGCAGGATTTCCACAGAAG and Rev CAGGTCACCACGTTTCTCTGCT. The qRT-PCR thermal cycling protocol included an initial denaturation step at 95 °C for 30 s, followed by 40 cycles of denaturation at 95 °C for 15 s and annealing at 60 °C for 60 s. A melting curve analysis was performed at the final dissociation stage, from 60 to 95 °C in 0.5 °C increments, to confirm specificity of the amplification products. Gene expression levels were normalized to β-actin as a housekeeping gene and expressed as fold-change relative to the corresponding solvent control.

### Quantification of Oxidative Stress Using High-Content Analysis

Intracellular, treatment-dependent oxidative stress was measured using HCA at two time points: in a short-term 0–2 h time series and after 48 h of exposure. H295R cells were seeded in black, clear-bottom 96-well plates at a density of 50,000 cells per well. For the short-term time series, medium was replaced after 24 h, and cells were incubated for an additional 48 h to replicate the conditions used in the steroidogenesis assay. Cells were then washed twice with PBS and stained with Hoechst 33342 (5 μM) and dihydroethidium (DHE, 15 μM) in FluoroBrite DMEM for 40 min. The fluorogenic probe DHE is hydroxylated by superoxide and other reactive oxygen species (ROS), forming 2-hydroxyethidium, which intercalates into DNA and emits a fluorescent signal at 606 nm.

After staining, cells in the short-term exposure group were washed with PBS and exposed to personalized POP mixtures, solvent control (DMSO, 0.1%), or positive control (Menadione, 100 μM) in FluoroBrite DMEM. Plates were imaged using a 10× objective at five sites per well with DAPI and Cy3 filters at 0, 0.5, 1, and 2 h using an ImageXpress Micro XLS Confocal High-Content Analysis System (Molecular Devices, Sunnyvale, CA, USA). For the 48 h time point, cells were seeded under identical conditions. After 24 h, the medium was replaced with POP mixtures or a solvent control. Following 48 h of exposure, cells were washed with PBS, stained, and imaged using the same protocol as for the short-term time series. Menadione (100 μM) was added 30 min before imaging as a positive control. Image analysis was conducted by using MetaXpress software with the integrated fluorescence application module to quantify fluorescence intensity. Average fluorescent nuclear intensity of DHE was calculated for each treatment condition based on six technical replicates from three independent experiments (*n* = 3).

### Statistical Analysis

To analyze the steroidogenesis and cell viability data and account for variability between experiments, a linear mixed model (LMM) was applied, with treatment concentration and mixture type as fixed effects and biological replicate (independent experiment) included as a random-effect term, using the lme4 package in Rstudio. Dunnett’s multiple comparison test was subsequently performed within the same package.^3^ Each mixture concentration group (1×, 10× and 100×) was compared with the corresponding solvent control. The analysis was performed on log-normalized data, as this improved compatibility with the LMM. Data points exceeding 2 standard deviations from the mean were considered outliers and removed before the analysis. The scripts used for statistical analyses are available at: https://github.com/flerpan01/POP-screening. Image analysis data were tested for statistical significance compared to solvent controls using one-way ANOVA with Dunnett’s multiple comparison test in GraphPad Prism version 8.4.3.

## Results

### Chemical-Class Submixtures Induced Unique Effects on Cellular Viability or Testosterone and Estradiol Synthesis in H295R Cells

Increased testosterone synthesis was observed at one or more concentrations in 5 of the 13 tested chemical-class submixtures, with 3 mixtures even inducing effects at the environmentally relevant 1× concentration (Figure 1 and Supplemental Figure S1). A slight decrease (5–7%) in cell viability was observed at all concentrations of the PCB submixture in PM#4, as well as the 10× concentration of the BDE submixture in PC1-OC-Mix (Figure 1 and Supplemental Figure S1 and Table S2). Estradiol was the least responsive end point, with only one submixture, at its highest tested concentration (100×, PCBs in PC1-OC-Mix), inducing a 23% increase in estradiol synthesis compared to the solvent control.

**Figure 1:**
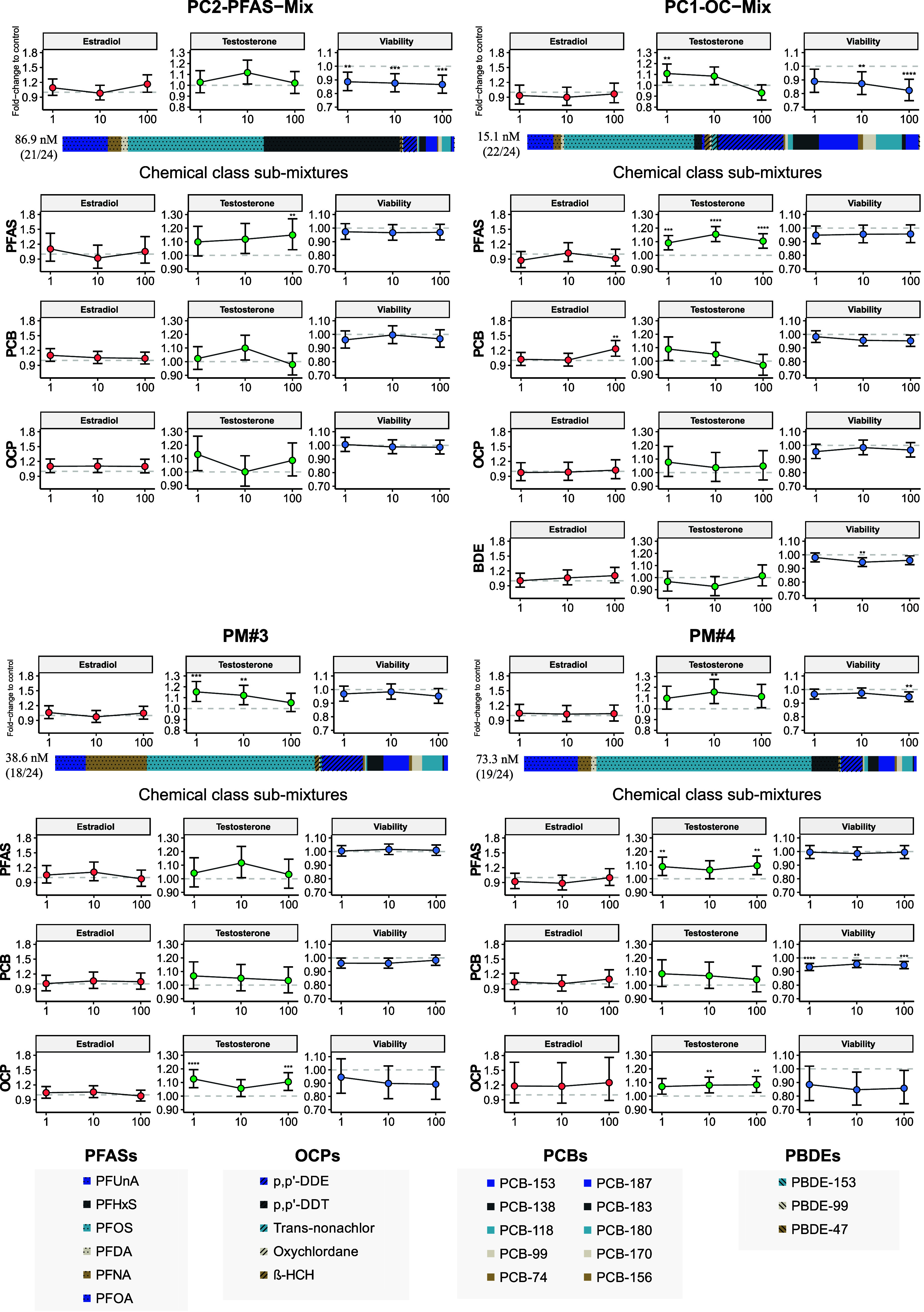
Effects of reconstructed personalized whole mixtures of POPs (PC1-OC-Mix, PC2-PFAS-Mix, PM#3, and PM#4) and their chemical-class submixtures on H295R cell viability, estradiol, and testosterone synthesis, after 48 h of exposure. Results are expressed as fold-change relative to the solvent control (0.1% DMSO), with error bars representing the 95% CI. The mixtures were tested at 1×, 10×, and 100× the concentrations detected in adult human blood plasma. Statistically significant differences from control are indicated as follows: *p < 0.05; **p < 0.01; ***p < 0.001; ****p < 0.0001 (LMM followed by Dunnett’s multiple comparison test).

Among the four tested PMs, only PC1-OC-Mix [68.2 nM] contained all six PFAS. The others lacked either PFUnA (PC2-PFAS-Mix [74.5 nM], PM#4 [58.5 nM]) or both PFUnA and PFDA (PM#3 [25.5 nM]) (Table S2). With the exception of the PFAS submixture in PM#3, which has the fewest components and the lowest concentration, the three other PFAS submixtures induced testosterone synthesis. In these active submixtures, testosterone levels increased by 9 to 15% compared to the solvent control (Table S1). Effects were evident at the 1× concentrations for PM#4 and PC1-OC-Mix, whereas the PFAS submixture of PC2-PFAS-Mix was only effective at the highest concentration (100×).

All four personalized whole mixtures contained the full set of 10 PCBs, though at different concentrations depending on the mixture. The two PCB submixtures with the highest total concentrations showed statistically significant effects on cell viability (PM#4, 1× to 100×, 7–5% decrease compared to control) and estradiol levels (PC1-OC-Mix, 100×, 23% increase). In contrast, the PCB submixtures with lower concentrations (PC2-PFAS-Mix and PM#3) did not affect any measured end point, even at 10 and 100×.

Both PC-2-PFAS-Mix and PC1-OC-Mix included all five OCPs, while PM#3 and PM#4 were lacking DDE or DDT, respectively. Two of the OCP submixtures induced small but statistically significant increases in testosterone synthesis at 1× for PM#3 (13%) and 10× for PM#4 (8%). No effects were observed for the OCP submixtures of PC2-PFAS-Mix or PC1-OC-Mix, which notably contained both the lowest (3.85 nM) and highest (30.4 nM) total concentration of the OCPs, respectively. The total concentrations of OCPs in the active submixtures of PM#3 and PM#4 were moderate and similar to each other (4.52–4.73 nM). These results indicate that the total OCP concentration alone is not the strongest predictor of effects on steroid hormone synthesis and rather that the mixture composition is the key factor. Notably, the active submixtures of PM#3 and PM#4 both lacked either DDE or DDT, while the submixtures that showed no effects included all five OCPs together. This may indicate that DDE and DDT, or the full combination of all five OCPs, could attenuate the biological response through antagonistic interactions.

Only one mixture, PC1-OC-Mix, contained more than one PBDE that could be tested as a submixture. The intermediate concentration of this submixture (10×, 5.9 nM) significantly reduced cell viability by 5%. However, this change was small compared to the solvent control, and the same effect was not observed at 100× (59 nM).

### Effects of Chemical-Class Submixtures Compared to the Corresponding Whole-Mixtures

For PM#3, it is evident from submixture testing that the OCPs drive the effect of the whole mixture (Figure 1), as a 15% increase in testosterone synthesis was observed at 1× of the whole mixture, closely mirroring the 13% increase in cells treated with the same concentration (1×) of the OCP submixture alone (β-HCH, oxychlordane, trans-nonachlor, and DDT). However, a minor discrepancy is observed at higher concentrations (Figure 1). While PM#3 as a whole mixture also induced testosterone synthesis at 10×, the OCP submixture stimulated testosterone at 100×, but not at the intermediate concentration of 10×. Although these changes are statistically significant, the magnitude of the increase is relatively small, ranging from 10 to 13% compared with the solvent control (Figure 1).

Similarly, for PM#4, the whole-mixture effect on testosterone synthesis (16% increase at 10×) could be partly attributed to the OCP submixture, which induced an 8% increase at the same concentration (Figure 1). As with PM#3, the effects of the OCP submixture do not strictly follow the same dose–response pattern as in the whole mixture. Statistically significant effects of the PM#4 OCP submixture were observed at both 10× and 100×, whereas the whole mixture only showed an effect at 10×. A similar trend was observed with the PFAS submixture of PM#4, which affected testosterone synthesis at 1× and 100×, but not at 10× (Figure 1). The whole PM#4 mixture also induced a small decrease (5%) in cell viability at 100× compared to the control. Similarly, a small but statistically significant decrease in viability (5–7%) was observed for the PM#4 PCB submixture at all three concentrations (1×, 10×, and 100×), in contrast to the whole mixture, which only reduced viability at the highest concentration.

In the whole-mixture tests of PC1-OC-Mix, H295R cell viability decreased by 13–18% at 10× and 100× compared to the solvent control. At 1× the mixture had no effect on viability, while the testosterone synthesis increased by 11%. Among the corresponding submixtures, the PFAS submixture induced a similar (9%) increase in testosterone synthesis at 1×, which likely explains the effect of the whole mixture. Notably, the PFAS submixture also induced testosterone synthesis at the higher concentrations (up to 15%), which was not evident in the whole mixture but might have been masked by the decreased viability at these concentrations (Figure 1, Table S2). Notably, only the PC1-OC-Mix submixture of PBDEs significantly reduced cell viability, and this effect was observed only at 10×, where viability decreased by 5% compared to the solvent control. This suggests that the PBDEs, potentially through interactions with other submixtures, may have contributed to the cytotoxicity observed in the whole mixture.

For the PC2-PFAS-Mix, cells exposed to the whole mixture at all three tested concentrations showed decreased viability (11–13%) compared to the control (Figure 1, Supplemental Figure S1 and Table S2). However, none of its chemical-class submixtures induced any statistically significant change in viability at any concentration, suggesting a toxicological interaction among chemicals from different classes.

### High-Content Screening Revealed No Effects of Personalized POP Mixtures on the Oxidative Stress Indicator DHE

In H295R cells exposed to whole personalized POP mixtures (PC1-OC-Mix or PC2-PFAS-Mix) for 0, 0.5, 1, and 2 or 48 h, neither of the mixtures induced oxidative stress, as measured by DHE oxidation. These mixtures were selected for testing based on their ability to decrease cell viability at low or medium concentrations. The positive control, menadione, significantly increased the nuclear DHE signal compared to the DMSO solvent control at 0.5, 1, and 2 h (Figure 2). A slight, nonsignificant decrease in signal from 0 to 2 h was observed in all samples except the positive control, likely due to photobleaching during the experimental procedure.

**Figure 2:**
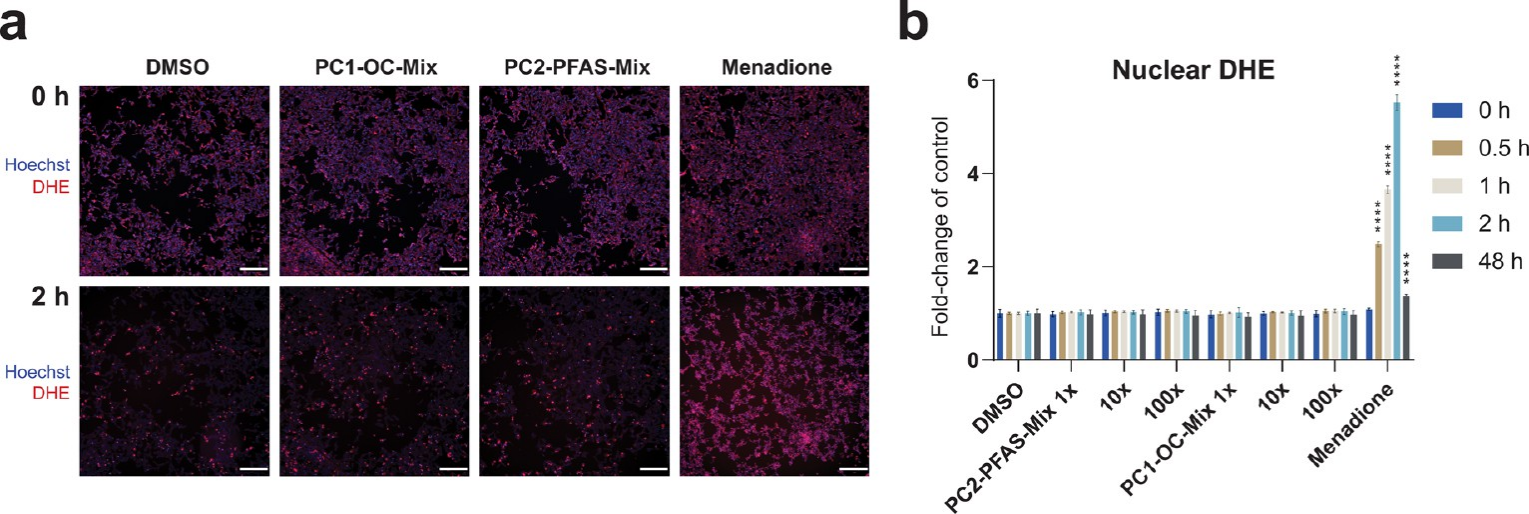
High-content imaging of H295R cells treated with solvent control (0.1% DMSO); the two reconstructed personalized whole mixtures of POPs (PC2-PFAS-Mix and PC1-OC-Mix) that decreased cell viability; or positive control (menadione, 100 μM) over a time series of 0, 0.5, 1, and 2 h after prestaining with Hoechst 33342 and DHE (a). Image analysis of the nuclear DHE stain intensity was conducted using MetaXpress. The average signal from three independent experiments (*n* = 3), each with six technical replicates, was calculated and visualized with standard deviation as error bars (b). Statistically significant differences from control are indicated as follows: ****p < 0.0001 (LMM followed by Dunnett’s multiple comparison test). Scale = 200 μm. None of the tested POP mixtures induced significant oxidative stress at any time point.

### Downregulation of the Rate-Limiting Steroidogenic Gene CYP11A1 After Exposure to Personalized Mixtures

In cells treated with the three whole personalized mixtures that affected testosterone levels, the highest concentration (100×) of PM#3 and PM#4 significantly downregulated *CYP11A1* expression to 45 and 53% of the solvent control levels, respectively. In contrast, the PC1-OC-Mix did not induce any changes in CYP11A1 expression. None of the tested POP mixtures affected *CYP19A1* (aromatase) expression at any concentration (Figure 3).

**Figure 3:**
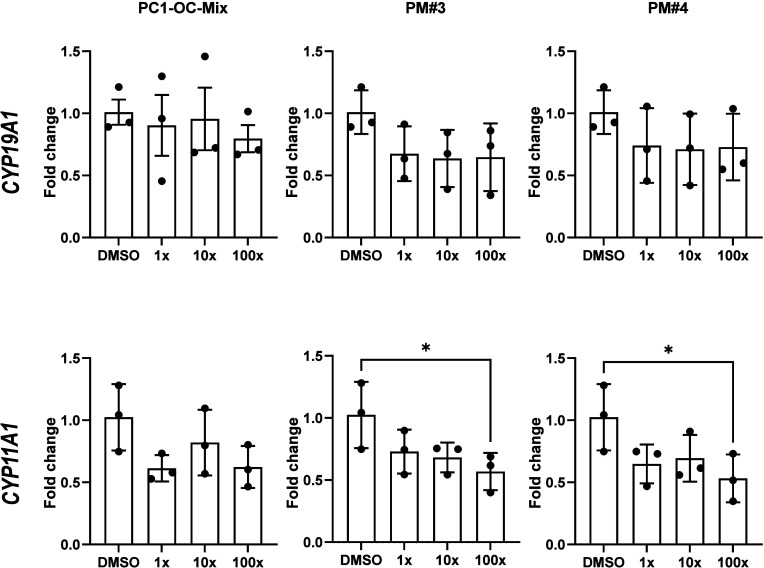
Gene expression of CYP19A1 (aromatase) and CYP11A1 in H295R cells exposed for 48 h to the three reconstructed personalized whole mixtures of POPs (PC1-OC-Mix, PM#3, and PM#4) that affected testosterone synthesis, analyzed by qPCR. Data represent the average fold-change compared to control (0.1% DMSO), summarized from three independent experiments (*n* = 3), each with technical triplicates. Error bars represent standard deviation. Statistically significant differences from control are indicated as follows: *p < 0.05; (ANOVA followed by Dunnett’s multiple comparison test).

## Discussion

In this study, we investigated the chemical drivers and underlying mechanisms of the personalized mixture effects on cell viability and sex hormone production in H295R cells. To accomplish this, we expanded on the previously developed mixture screening strategy for assessing steroidogenic effects, specifically on testosterone and estradiol, as well as cell viability, reported in our recent studies.^3,10^ The mixtures were separated into chemical classes (PFASs, PCBs, OCPs, and PBDEs) to investigate the contribution of the submixtures and identify major drivers of the effects observed in the whole mixtures. Several, but not all, of the effects induced by the personalized POP mixtures could be attributed to a specific chemical class when tested as submixtures. However, not all submixture effects translated directly to those observed in the full mixtures, as discussed further below.

Exposure to complex chemical mixtures is a significant public health concern. Endocrine disruption is linked to infertility as well as several major noncommunicable diseases, including cancer, metabolic disorders, and cardiovascular disease (CVD). Mixtures reconstructed from blood levels of three individuals from the Swedish VIP cohort, a health program established to address the region’s elevated CVD rates,^21^ increased the testosterone levels at both the 1× (11–15%) and 10× concentrations (12–16%). In addition to traditional risk factors, such as high-calorie diet, alcohol use, smoking, physical inactivity, and genetic susceptibility, growing evidence indicates that chemical exposures, even during prenatal life, may contribute to metabolic syndrome and CVD.^23–28^ Research has linked exposure to POPs, at levels comparable to those in our study, with increased CVD mortality in a Swedish population.^29,30^ Disruption of sex hormone signaling may be one of the underlying mechanisms, as both estrogen and testosterone play critical roles in cardiovascular physiology.^31^ For example, testosterone can exacerbate neutrophil-driven inflammation and worsen cardiac injury following myocardial infarction, which may help explain why men often develop larger infarct sizes than women.^32,33^

Identifying the main compounds contributing to environmentally relevant chemical stress remains a major challenge.^11,34^ Shifting the regulatory paradigm from classical single-compound assessments to a more holistic mixture toxicity approach is critical for next-generation risk assessment.^34,35^ As recently demonstrated, a personalized mixture toxicology approach can improve our understanding of interindividual differences in chemical exposure and response.^10^ Grouping chemicals by class for risk assessment purposes has been suggested as a practical strategy to simplify both experimental testing and the regulatory evaluation of complex mixtures.^36^ A class-based approach can also inform experimental design by helping identify which chemical groups warrant focused testing, enabling more efficient mixture reconstruction and providing insight into key drivers of biological effects. However, our findings demonstrate the important limitations of relying solely on class-based grouping. The chemical-class submixtures of four personalized POP mixtures induced effects that, in some cases, explained those observed for the complete POP mixtures. For example, the OCP submixture of PM#3 increased testosterone synthesis by 13% at 1× concentration, similar to the 15% increase observed with the full mixture. Likewise, the OCP submixture of PM#4 accounted for approximately half of the 16% testosterone increase observed with the full mixture at 10× concentration. The PFAS submixture from PM#4 also induced testosterone synthesis, but only at 1× and 100× concentrations, differing from the 10× concentration that produced effects in the whole mixture. Importantly, several chemical-class submixtures induced statistically significant effects that were not observed in their corresponding whole mixtures. For example, the PFAS submixture of the PC2-PFAS-Mix increased testosterone synthesis by 15% at 100×, an effect not observed in the whole mixture containing PCBs and OCPs. A similar pattern was observed for estradiol, where a 23% increase was induced by the PCB submixture of the PC1-OC-Mix at 100×, but not by the whole mixture. These results suggest that additive assumptions within chemical classes do not always hold and that interactions between classes can alter the overall biological effect. Among the OCP submixtures, only those lacking either DDE (PM#3) or DDT (PM#4) increased testosterone synthesis in H295R cells, whereas OCP submixtures with both lower and higher total concentrations induced no effect. This may indicate antagonistic effects within more complex mixture compositions, independent of total concentration, where individual effects of the components are canceled out. Previous studies have shown that OCP mixtures can induce antagonistic effects on voltage-gated calcium channel (VGCC) inhibition, CYP2B6 activity, cytotoxicity, and genotoxicity.^37–40^ In contrast, the PC2-PFAS-Mix reduced cell viability at all concentrations, while none of its submixtures induced such effects, suggesting that interactions within the whole mixture may enhance cytotoxicity. Several other studies have reported that chemicals can produce effects that are either lost or only become evident when combined with others.^41–44^ For example, the PCB-180 metabolite 3′–OH-PCB-180 has been shown to enhance benzo­[a]­pyrene (BaP) genotoxicity in HEPG2 cells through induction of CYPA1, a BaP-activating enzyme.^45^ Similarly, pre-exposure to DDT increases the toxicity of chlordane in rats by altering metabolic enzyme profiles.^46^ In addition, DDT can affect the activity of membrane transporters, leading to potential toxicokinetic interactions.^38^ Taken together, these findings highlight that although class-based grouping can be a useful tool for structuring mixture research and regulatory assessment, it cannot substitute for studies that evaluate realistic multichemical exposures. Understanding how chemical classes interact within person-specific exposome-based mixtures is therefore important for accurately assessing human health risks. Beyond altered metabolism and interactions with the same biological target, synergistic, agonistic, and antagonistic mixture effects may also arise from mechanisms such as modulation of efflux transporters, changes in membrane permeability, or altered receptor expression.^47^ PFASs, for example, can integrate into cellular membranes, modify their permeability, and bind to nuclear receptors and transport proteins.^48,49^ The use of acoustic liquid handling in this study enabled precise and adaptable mixture generation. Future studies investigating the role of specific contributors in POP mixtures could involve more comprehensive subtractive screening, where individual compounds are systematically removed to assess their specific contributions.

Oxidative stress is a well-established mechanism through which toxicants can impair both steroid hormone synthesis and cell viability.^50–53^ It was therefore investigated as a potential contributor to the effects observed in response to the personalized POP-mixture exposure. HCA was used to measure oxidative stress in a setup that also enabled the collection of culture media for hormone analysis from the same cells, thereby optimizing the workflow and analytical efficiency. Using the fluorogenic probe DHE, we did not detect any induction of oxidative stress in cells exposed to either of the two tested POP mixtures. These findings suggest that oxidative stress is unlikely to account for the observed disruptions in cell viability and steroidogenesis. However, additional experiments using complementary markers for oxidative stress and mitochondrial health are warranted to confirm this observation.

Some of the whole and submixtures showed patterns consistent with nonmonotonic dose–response relationships, a well-established phenomenon for endocrine-disrupting chemicals in which toxicity does not necessarily follow a traditional sigmoidal concentration–response curve.^54,55^ In such cases, adverse effects may occur at low concentrations that cannot be predicted by extrapolating them from higher concentrations. The lack of effects on testosterone production at the highest concentrations tested in this study could, for example, reflect antagonistic interactions at high concentrations or saturation of the relevant biological targets. Other possible explanations include reduced formation of active metabolites as cellular metabolic capacity becomes limited or the activation of compensatory mechanisms once a certain threshold is reached. Investigation of mixture-induced effects on key steroidogenic enzymes may help elucidate the mechanistic basis for these nonlinear dose–response patterns. At the highest concentration (100×), the PM#3 and PM#4 mixtures decreased *CYP11A1* expression, coinciding with a return of testosterone concentration to control levels, following the increases observed at lower concentrations. This pattern may indicate a compensatory mechanism in which the rate-limiting step in steroidogenesis is downregulated to counteract the elevated level of testosterone synthesis. Previous research has shown similar feedback inhibition of *CYP11A1* by testosterone both *in vitro* and *in vivo*.^56^ In contrast, the expression of *CYP19A1* (aromatase), which converts androstenedione to estrone and testosterone to estradiol, remained unchanged. Future studies should examine additional enzymes in the steroidogenic pathway. Androgens are known to suppress other key steroidogenic regulators such as the cholesterol transport protein StAR, which facilitates mitochondrial cholesterol import.^57^ Time-course analyses of gene expression, protein abundance, and hormone levels, together with enzymatic activity, would help clarify the temporal sequence of these feedback mechanisms and determine whether the observed gene-expression changes translate into functional alterations in steroidogenesis.

Given that the studied mixtures reflect real-world human exposure patterns, the findings have environmental implications by highlighting the potential effects of background POP levels. The work is also relevant for regulatory efforts aimed at improving mixture risk assessment. However, several limitations of this study should be considered when interpreting the findings. Although the personalized POP mixtures induced reproducible changes in cytotoxicity and steroidogenesis at levels detected in individuals’ blood, the human relevance of these effects likely depends on several factors. While the observed changes in hormone levels were relatively small (11–16%), the endocrine system is highly sensitive to disruption, and adverse effects driven by such changes cannot be excluded.^54,55^ Furthermore, endocrine and cytotoxic pathways *in vivo* are considerably more complex, and additional end points such as broader transcriptomic responses, epigenetic alterations, receptor activation assays, or long-term functional measures were beyond the scope of the study. It should also be noted that the reconstructed mixture concentrations were based on measured plasma levels, which may not fully reflect the bioavailable or intracellular concentrations *in vivo*. In addition, biological variability, including interindividual differences in metabolism, distribution, steroidogenic enzyme, and receptor expression, could modulate the responses. Moreover, while comparisons between whole mixtures and submixtures suggested potential interactions, including antagonistic effects among OCPs, these interpretations were not tested against formal mixture-interaction models such as concentration addition or independent action. However, recent studies in zebra fish have shown similar effects, in which individual compounds may antagonize the activities of other chemicals, and submixtures may either enhance or suppress the effects of others.^44^ Follow-up studies with expanded mechanistic depth, including subtractive screening, “add-back” or ratio-manipulation experiments, are warranted to more fully elucidate the drivers and modes of action of real-world POP mixtures.

Taken together, our findings show that personalized POP mixtures can affect steroid hormone synthesis in H295R cells, with some chemical class-specific submixtures reproducing the effects of full mixtures on testosterone levels, while others induced distinct changes in cell viability and hormone synthesis. These observations highlight the complexity of mixture interactions and suggest that antagonistic effects may reduce certain responses when multiple components are combined at different concentrations. Downregulation of CYP11A1 may contribute to the observed changes in testosterone synthesis, although further studies are required to confirm this hypothesis. HCA in a setup complementary to the downscaled OECD TG#456 steroidogenesis assay, indicated that mechanisms other than oxidative stress drive the mixture-induced reduction in cell viability. Overall, these findings, together with the novel approaches used here, underscore the importance of evaluating individual chemical mixture effects under realistic exposure conditions (i.e., realistic concentrations and realistic mixture compositions) using automated assay platforms to better inform mixture health risks and support improved regulatory assessment.

## Supplementary Material



## Notes

Figures and tables detailing the mixture compositions and results of the H295R steroidogenic screening (PDF)
