# Supplementary material for: Chemical-Class Submixture Screening Reveals Drivers of Endocrine Disruption in Personalized Human Blood POP Mixtures
Source: Environ Sci Technol. 2026 Feb 3;60(6):4648–57. doi: 10.1021/acs.est.5c13521 (PMC12918528; doi:10.1021/acs.est.5c13521)
Supplement: Supplementary file 1 [file es5c13521_si_001.pdf]

SUPPORTING INFORMATION

Chemical-class sub-mixture screening reveals drivers of endocrine disruption in personalized human blood POP mixtures

Denise Strand[1], Paula Pierozan[1], Luã Reis[1], Bo Lundgren[2], Jonathan W. Martin[1], Oskar Karlsson[1*]

[1]Science for Life Laboratory, Department of Environmental Science, Stockholm University, Stockholm, 114 18, Sweden

[2]Science for Life Laboratory, Biochemical and Cellular Assay unit, Dept. of Biochemistry and Biophysics, Stockholm University, Stockholm, 106 91, Sweden

* Corresponding author: Oskar Karlsson, Science for Life Laboratory, Department of Environmental Science, Stockholm University, 11418, Stockholm, Sweden.

E-mail: Oskar.Karlsson@aces.su.se

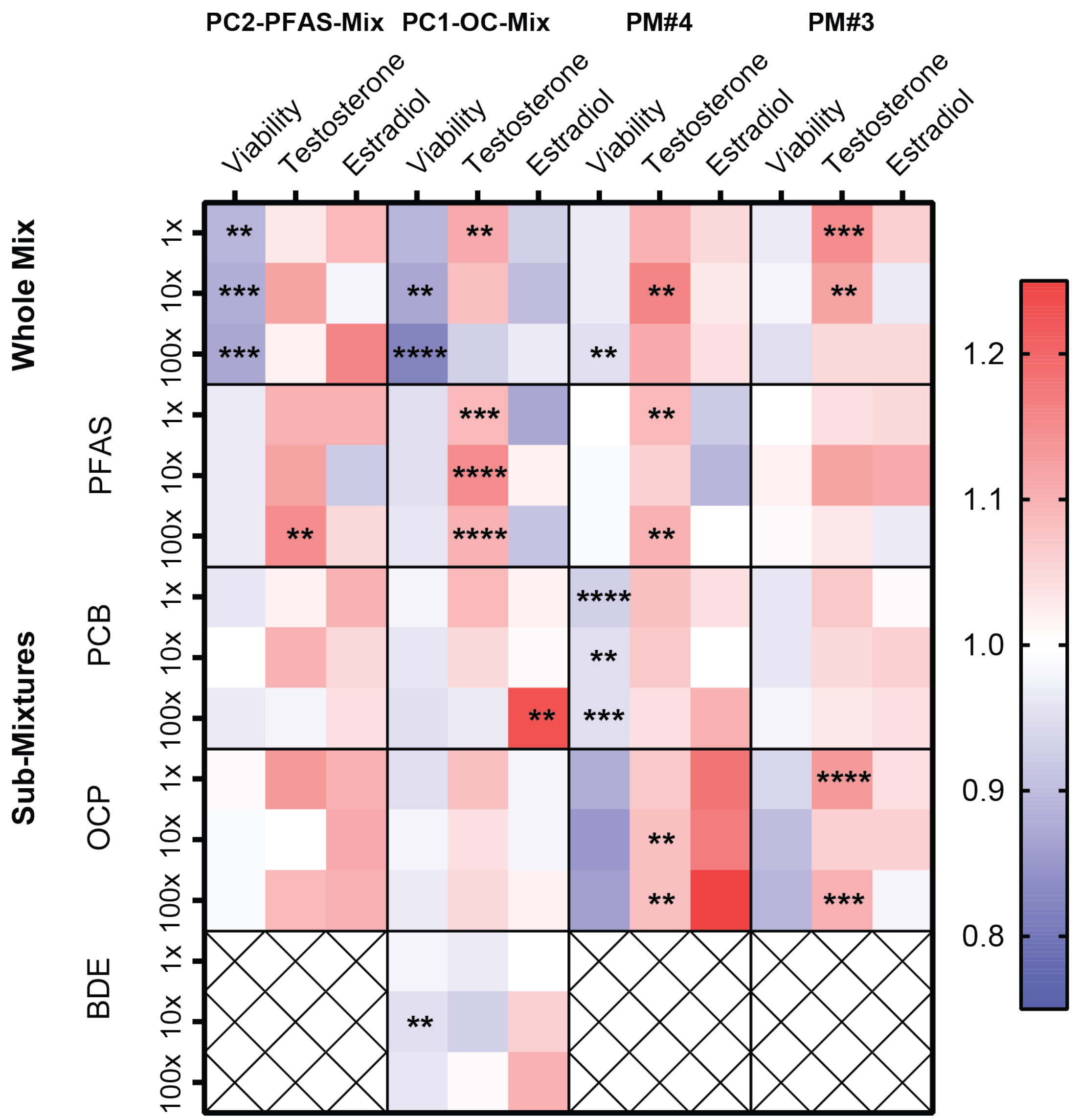

Figure S1. *Heatmap of fold-change in viability, and synthesis of testosterone and estradiol in H295R cells treated with whole personalized mixtures and their sub-mixtures divided by chemical class (PFAS, PCB, OCP, BDE) at three concentrations (1×, 10× 100×). Statistical significant changes from solvent control are indicated with stars (*p < 0.05; **p < 0.01; ***p < 0.001; ****p < 0.0001, based on a LMM followed by Dunnett's multiple comparison test.*

***Table S1**. Composition of compound class POP sub-mixtures separated into PFAS, OCPs, PCBs and PBDEs*

			**Reconstructed personalized mixtures**			
**Compound class**	**Compound**	**CAS**	**PC2-PFAS**	**PC1-OC**	**PM#3**	**PM#4**
**PFAS**	PFOA	335-67-1	1E-08	1E-08	3E-09	1E-08
	PFNA	375-95-1	3E-09	3E-09	6E-09	2.5E-09
	PFDA	335-76-2	1.5E-09	1.2E-09	N/A	1E-09
	PFOS	2795-39-3	3E-08	5E-08	1.5E-08	4E-08
	PFHxS	82382-12-5	3E-08	3E-09	1.5E-09	5E-09
	PFUnA	2058-94-8	N/A	1E-09	N/A	N/A
**Total PFAS concentration (M)**			**7.45E-08**	**6.82E-08**	**2.55E-08**	**5.85E-08**
**OCPs**	β-HCH	319-85-7	5E-10	2E-09	4E-10	3E-10
	Oxychlordane	27304-13-8	8E-11	8E-10	1.3E-10	7E-11
	Trans-nonachlor	39765-80-5	1.5E-10	2E-09	2E-10	1.5E-10
	p,p'-DDT	50-29-3	1.2E-10	6E-10	4E-09	N/A!
	p,p'-DDE	72-55-9	3E-09	2.5E-08	N/A	4E-09
**Total OCP concentration (M)**			**3.85E-09**	**3.04E-08**	**4.73E-09**	**4.52E-09**
**PCBs**	PCB-74	32690-93-0	1.5E-10	7E-10	2E-10	1.5E-10
	PCB-99	38380-01-7	1.5E-10	1E-09	1E-10	2.5E-10
	PCB-118	31508-00-6	3E-10	2E-09	1.5E-10	7E-10
	PCB-138	35065-28-2	1.5E-09	1E-08	1.6E-09	2E-09
	PCB-153	35065-27-1	2.5E-09	1.5E-08	2.5E-09	3E-09
	PCB-156	38380-08-4	3E-10	2E-09	3E-10	4E-10
	PCB-170	35065-30-6	8E-10	5E-09	1E-09	1E-09
	PCB-180	35065-29-3	2E-09	1E-08	2E-09	2E-09
	PCB-183	52663-69-1	2E-10	1.5E-09	1.5E-10	2.5E-10
	PCB-187	52663-68-0	6E-10	5E-09	4E-10	5E-10
**Total PCB concentration (M)**			**8.50E-09**	**5.22E-08**	**8.40E-09**	**1.03E-08**
PBDEs	PBDE-47	5436-43-1	5E-11	4E-10	N/A	N/A
	PBDE-99	60348-60-9	N/A	4E-11	N/A	N/A
	PBDE-153	68631-49-2	N/A	1.5E-10	N/A	N/A
**Total PBDE concentration (M)**			**5E-11**	**5.90E-10**	**N/A**	**N/A**
**Whole-mixture concentration (M)**			**8.69E-08**	**1.51E-07**	**3.86E-08**	**7.33E-08**

Table S2. Summary of results from mixture-screening of effects on testosterone-and estradiol synthesis and viability in H295R cells.

		Estradiol			Testosterone			Viability		
		FC	LMM	Dun	FC	LMM	Dun	FC	LMM	Dun
**PM#3** PFAS	1×	1.05		0.98092	1.04		0.93075	1.00		0.99987
	10×	1.11	0.44666	0.69950	1.12	0.19361	0.16209	1.02	0.86209	0.91655
	100×	0.97		0.99925	1.03		0.97813	1.01		0.99175
**PM#3** PCB	1×	1.01		1.00000	1.07		0.56355	0.96		0.19715
	10×	1.06	0.85673	0.94787	1.05	0.54835	0.79836	0.96	0.12212	0.18524
	100×	1.04		0.98588	1.03		0.95536	0.98		0.88298
**PM#3** OCP	1×	1.04		0.94783	1.13		0.00044	0.94		0.91947
	10×	1.06	0.49254	0.84815	1.06	0.00038	0.28343	0.90	0.32615	0.45849
	100×	0.98		0.99721	1.10		0.00537	0.89		0.39535
**PM#4** PFAS	1×	0.92		0.83688	1.09		0.03560	1.00		0.99997
	10×	0.89	0.39316	0.54093	1.06	0.01430	0.21196	0.99	0.94390	0.97847
	100×	1.00		1.00000	1.10		0.01903	0.99		0.99993
**PM#4** PCB	1×	1.04		0.99496	1.08		0.33452	0.93		0.00001
	10×	1.00	0.65422	1.00000	1.07	0.32202	0.53410	0.95	0.00001	0.00677
	100×	1.10		0.75293	1.04		0.89789	0.95		0.00080
**PM#4** OCP	1×	1.18		0.85582	1.07		0.06830	0.88		0.35982
	10×	1.17	0.61572	0.87061	1.08	0.00990	0.02566	0.85	0.08651	0.10535
	100×	1.25		0.65052	1.08		0.01925	0.86		0.15830
**PC1-OC** PFAS	1×	0.87		0.53156	1.09		0.00225	0.95		0.45556
	10×	1.02	0.29009	0.99986	1.15	0.0000001	0.00000	0.95	0.39812	0.60470
	100×	0.91		0.84366	1.10		0.00026	0.96		0.64147
**PC1-OC** PCB	1×	1.02		0.99908	1.09		0.16467	0.98		0.93764
	10×	1.01	0.00264	1.00000	1.05	0.02152	0.68653	0.96	0.08765	0.19508
	100×	1.23		0.00766	0.97		0.93752	0.95		0.13279
**PC1-OC** OCP	1×	0.98		0.99976	1.08		0.55073	0.95		0.36415
	10×	0.98	0.95935	0.99998	1.04	0.56347	0.96237	0.98	0.34825	0.98066
	100×	1.02		0.99975	1.05		0.88209	0.97		0.66325
**PC1-OC** BDE	1×	1.00		1.00000	0.97		0.93754	0.98		0.72969
	10×	1.06	0.44872	0.92702	0.93	0.16034	0.32250	0.95	0.00675	0.00654
	100×	1.10		0.56712	1.01		0.99896	0.96		0.07715
**PC2-PFAS** PFAS	1×	1.10		0.93764	1.10		0.26382	0.97		0.87996
	10×	0.92	0.52525	0.96549	1.12	0.03468	0.12425	0.97	0.65184	0.74539
	100×	1.05		0.99743	1.15		0.03034	0.97		0.80270
**PC2-PFAS** PCB	1×	1.10		0.38118	1.02		0.98629	0.96		0.69221
	10×	1.05	0.42492	0.89262	1.10	0.03667	0.12049	1.00	0.53355	1.00000
	100×	1.04		0.96148	0.98		0.98859	0.97		0.84411
**PC2-PFAS** OCP	1×	1.10		0.46885	1.13		0.15837	1.01		0.99988
	10×	1.11	0.32066	0.43263	1.00	0.08165	1.00000	0.99	0.85385	0.99559
	100×	1.10		0.50301	1.09		0.53530	0.99		0.98708

Results are presented as fold-change of control (FC), and statistical significance (α=0.05) based on a liner mixed-effects model (LMM) and a Dunnett's multiple correction post-hoc test (Dun) is indicated.
